# Measuring inter- and intra-individual differences in visual scan patterns in a driving simulator experiment using active information storage

**DOI:** 10.1371/journal.pone.0248166

**Published:** 2021-03-18

**Authors:** Christiane B. Wiebel-Herboth, Matti Krüger, Patricia Wollstadt

**Affiliations:** Honda Research Institute Europe, Offenbach/Main, Germany; Tongii University, CHINA

## Abstract

Scan pattern analysis has been discussed as a promising tool in the context of real-time gaze-based applications. In particular, information-theoretic measures of scan path predictability, such as the gaze transition entropy (GTE), have been proposed for detecting relevant changes in user state or task demand. These measures model scan patterns as first-order Markov chains, assuming that only the location of the previous fixation is predictive of the next fixation in time. However, this assumption may not be sufficient in general, as recent research has shown that scan patterns may also exhibit more long-range temporal correlations. Thus, we here evaluate the active information storage (AIS) as a novel information-theoretic approach to quantifying scan path predictability in a dynamic task. In contrast to the GTE, the AIS provides means to statistically test and account for temporal correlations in scan path data beyond the previous last fixation. We compare AIS to GTE in a driving simulator experiment, in which participants drove in a highway scenario, where trials were defined based on an experimental manipulation that encouraged the driver to start an overtaking maneuver. Two levels of difficulty were realized by varying the time left to complete the task. We found that individual observers indeed showed temporal correlations beyond a single past fixation and that the length of the correlation varied between observers. No effect of task difficulty was observed on scan path predictability for either AIS or GTE, but we found a significant increase in predictability during overtaking. Importantly, for participants for which the first-order Markov chain assumption did not hold, this was only shown using AIS but not GTE. We conclude that accounting for longer time horizons in scan paths in a personalized fashion is beneficial for interpreting gaze pattern in dynamic tasks.

## Introduction

In the context of driving, visual scanning behavior must be organized such as to obtain relevant information for the driving task just in time. This organization includes not only choosing the right location but also the right order and timing of fixations for extracting visual information. Quantifying the intra- and inter-individual variance of this scanning behavior might have the potential to discover driving-relevant attributes, such as changes in driver state [1], but also individual driver characteristics. As a consequence, the analysis of visual scanning behavior might allow for a detailed characterization of individual driving behavior, and eventually the fine-tuning of adaptive driver assistance systems. In the context of human-vehicle cooperation, the development of driving assistance systems that can adapt its functionalities in order to support the driver in demanding situations or according to the driver’s individual needs has been put forward as an important topic, lately [2]. For such adaptations to be useful one important prerequisite is usually to identify relevant changes in user states or user behavior and to evaluate the effects of the adaptation on the user. In the context of driving, physiological markers such as EEG (e.g., [3]), fMRI (e.g., [4]), gaze behavior (e.g., [5]) or heart rate (e.g., [6]) as well as measures of the driving behavior (e.g., [7, 8]) have been investigated among others for assessing the driver state or to evaluate the impact of contextual changes. Chen et al. [4] for example investigated the effect of decorated side walls in tunnels on the driver. Here, we want to extend previous research on the potential of visual scanning behavior as an unobtrusive measure for assessing differences in driver states and driving behavior.

In the analysis of visual scanning behavior, information-theoretic measures have been introduced as a powerful tool to detect changes in observer states. For example, the gaze transition entropy (GTE) has been introduced to quantify the regularity of scan patterns [9, 10] and has been shown to change under variations in task demand or observer state (see [11] for a review). However, the GTE assumes that fixation sequences can be sufficiently modeled by a Markov chain of order one, such that the location of a fixation depends only on its immediate past fixation, while dependencies over longer time horizons are not accounted for. However, recent findings suggest that including such long-range dependencies when modeling scanning behavior is beneficial in describing individual viewing behavior [12–14].

In the present work, we therefore evaluate an alternative information-theoretic approach for quantifying scan path regularity in a dynamic driving task, the active information storage (AIS) [15]. In contrast to the GTE, the AIS optimizes the time span of past fixations being informative for the predictability of the next fixation and thus entering the computation of scan path regularity. To evaluate whether this representation of scan paths is beneficial in the context of human state estimation in a dynamic task like driving, we compare the two measures on data from a driving simulator experiment.

### Related work

Visual scanning behavior is shaped by both bottom-up information such as low-level image features, as well as top-down processes such as high-level cognitive systems or prior knowledge that modulate gaze behavior to solve a task at hand (e.g., [16–18]). In recent years, the study of sequences of fixations or *scan paths*, as one aspect of viewing behavior, has substantially gained interest [16, 19]. One popular tool for quantitatively describing scan paths as well as their intra- and inter-individual differences, are entropy measures [11], in particular the stationary gaze entropy (SGE) and the gaze transition entropy (GTE) [9, 10]. The SGE is a measure of overall spatial dispersion of gaze locations in a given time window, where high values of SGE indicate a highly equiprobable distribution of gaze points across all predefined areas of interest (AOI), and low values indicate a more concentrated distribution of gaze points. On the other hand, the GTE is defined as the entropy of the gaze transitions within a given time window. Low GTE values indicate a high regularity in gaze transitions and high values indicate more random or erratic transitions [10]. The GTE has also been interpreted as the overall predictability of scan patterns [11].

The SGE and GTE were first systematically applied to scan path data by Krejtz et al. to compare scan paths between individuals viewing different types of art works [9, 10]. Further studies have successfully applied both measures, in particular to detect changes in user states, e.g., due to differences in workload [20], drowsiness [5], alcohol consumption [11], or increased levels of anxiety [21, 22] (for a review see Shiferaw et al. [11]). Measures have also been used to evaluate teaching [23] or advertisement material [24], and have been proposed as potential online markers for attentional shifts in driving [1].

Importantly, existing information-theoretic approaches to scan path analysis such as the GTE, assume that scan paths can be modelled as Markov chains of order one, such that the probability of the next gaze point only depends on the single prior gaze point in time, while all earlier fixations do not exhibit an influence on future gazing behavior. This assumption is often not tested in studies employing the GTE and has been challenged by a series of more recent findings. Krejtz et al. [9, 10] explicitly tested the Markov assumption based on a procedure proposed by Besag and Mondal [25] and found that the assumption was valid for most participants, but not for all. A number of studies using modelling approaches other than information-theoretic measures, e.g., successor representation models from reinforcement learning, have found evidence for a longer lasting influence of past fixations on future gaze behavior [12, 13, 19, 26]. In particular, individual differences in gaze behavior were shown to be better reflected when accounting for a longer temporal horizon in scan paths [12, 13]. Such an approach allowed for example to explain up to 40% of the variance in viewer intelligence, working memory capacities, and speed of processing [12, 19]. Moreover, Hoppe et al. [27] provided first quantitative evidence that humans are capable of planning eye movements beyond the next fixation.

### Current study

In sum, previous findings suggest that more long-term correlations in scan paths exist, and challenge the assumption that scan paths can be sufficiently modelled by first-order Markov chains. As a result, existing approaches making use of this assumption, may underestimate the true regularity of gaze patterns and may fail to estimate the total predictability of scan paths. The resulting non-optimal assessment of a scan path’s predictability may in turn lead to erroneous conclusions about changes in gaze behavior. Such misclassifications may have an especially detrimental effect when using entropy measures in gaze-based applications, for example, driving assistance [1, 28]. Therefore, we here propose to extend existing information-theoretic approaches to quantitative scan path analysis by both, testing and accounting for long-term dependencies in gaze behavior.

In particular, we set out to test the following hypotheses:

1. H1: In a dynamic task such as driving, the assumption of scan paths being sufficiently described by a first-order Markov chain is not generally valid, instead, fixations can exhibit a longer-lasting influence on gaze patterns.
2. H2: Individuals can differ in the time horizon of past fixations being predictive for future gaze behavior.
3. H3: An individually optimized scan path representation accounting for potential long-range dependencies in gaze behavior will result in a better distinctiveness of different user states.

To test our hypotheses, we here use a novel approach for measuring the predictability of scan paths by estimating the AIS [14, 15] using a recently proposed estimation algorithm [29, 30]. AIS quantifies the predictability of a time series by measuring how much information the past of a sequence provides about its next state and has been successfully applied in a variety of applications, e.g., complex systems analysis [31], neuroscience [32], and biology [33]. The applied estimation algorithm optimizes the past state used for AIS calculation in a data-driven fashion and thus allows to account for long-range temporal dependencies as well as inter-individual differences.

We apply the proposed approach to gaze data recorded during a driving simulator experiment and evaluate whether both GTE and AIS are equally sensitive to changes in driver viewing behavior during different driving tasks and difficulties. Supporting our hypotheses, our results suggest that in some participants the general assumption of scan paths following a first-order Markov chain does not hold, and that for these participants, the GTE is not as sensitive to changes in scan path predictability as is the AIS. As a result, we propose AIS as a promising tool in the description of viewing behavior, which may have the potential to discover changes in driver state that are relevant in gaze-based applications [1, 11], and may be missed by existing scan path measures.

Prior evidence that AIS is suitable for detecting changes in user state, was recently provided in a proof-of-concept study, where we estimated AIS from eye tracking data recorded during a simple visual search task under two experimental manipulations intended to vary user states [14]. We here extend this work by directly comparing AIS and GTE in their ability to detect changes in user states during a dynamic task.

## Materials and methods

The data reported here were recorded as part of a larger study. Details on the entire experiment can be found in [34, 35].

### Participants

13 participants took part in the entire experiment (12 males, mean age 33 [24 to 43]) [34, 35]. Nine of the participants were categorized as highly experienced drivers while four were categorized as little-to medium experienced drivers based on the driven kilometers per year. Two participants were excluded from the analysis reported here due to technical failures during the eye tracking recording. The experiment was approved by our Institutional Review Board (HG Bioethics Committee) in line with the Declaration of Helsinki. All participants gave their written informed consent before taking part in the experiment. All participants had normal or corrected to normal vision.

### Apparatus

The experiment took place in a static driving simulator running SILAB 5.1 (WIVW GmbH) with real-vehicle controls for steering, braking and accelerating. The setup comprised three display panels (50 inch diagonal, Resolution: 3 x 1080p, updated at 60 Hz) that offer approximately a 160° field of view. Front, side, and rear mirrors, as well as a simplified dashboard were rendered into the driving scene.

**Fig 1:**
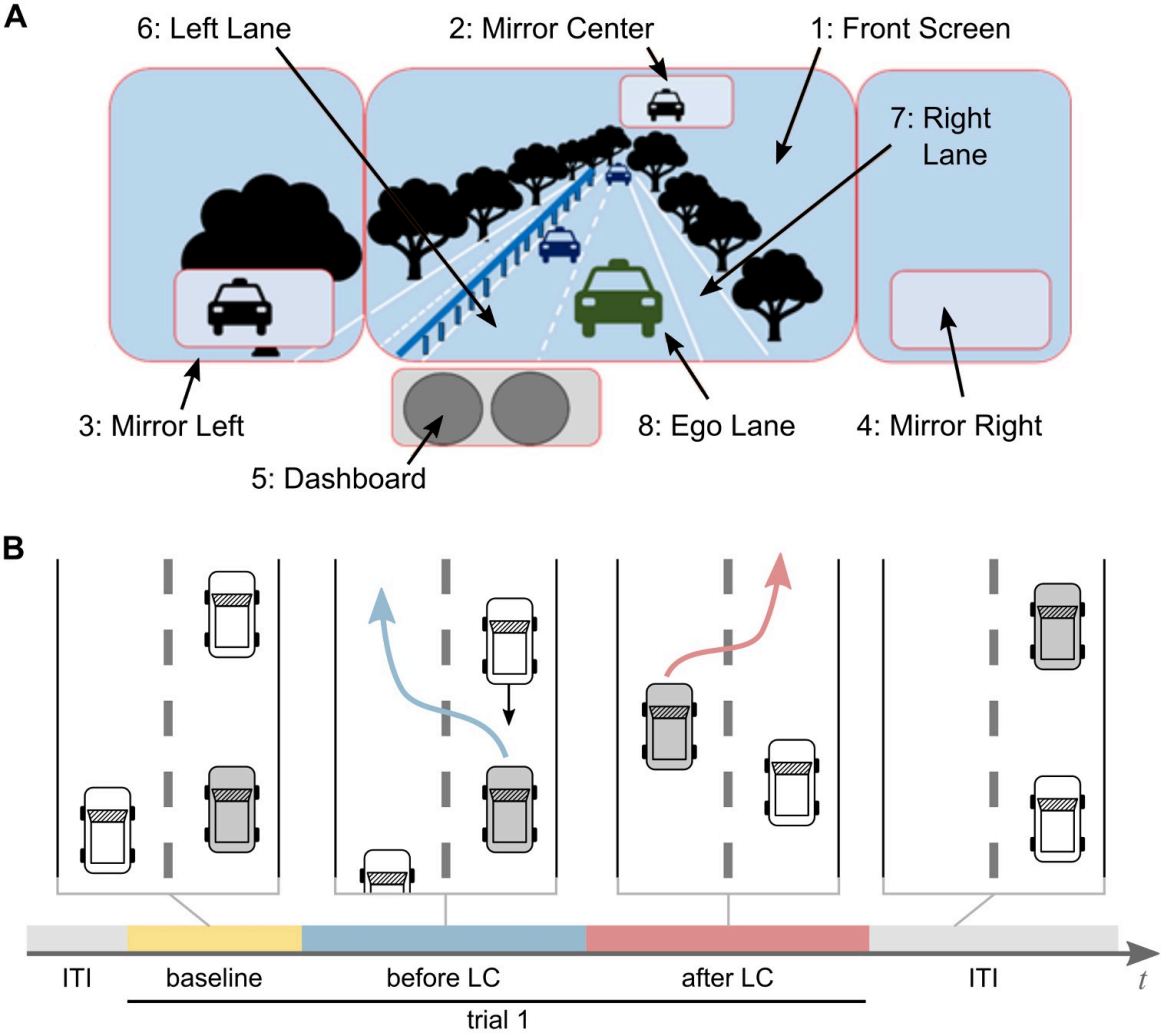
Definition of areas of interest (AOI) and experimental procedure. (A) AOI defined based on their semantic meaning in the driving task. (B) Experimental procedure: Each trial comprises a *baseline* period of five fixations and the task interval that starts with the deceleration of the car in front of the ego-car (grey). The task is split into the period *before lane change*, between trial onset and changing onto the left lane (blue arrow and marker), and the period *after lane change*, between changing onto the left lane and changing back onto the right lane (red arrow and marker).

The participant’s gaze behavior was recorded using a Pupil Labs eye tracker (Pupil Labs GmbH, see [36]), using 120 Hz monocular eye tracking and 60 fps world camera recordings. Gaze points were mapped to eight AOI on the simulator screens via the screen marker solution implemented by Pupil Labs. AOI were defined based on their semantic meaning in the task, including the rear mirror, the left side mirror, the right side mirror, the dashboard speedometer, the ego lane in front of the ego car, the left lane in front of the ego car, the right lane in front of the ego car and the remaining front shield view (Fig 1A). The eye tracker was calibrated before the start of each experimental session. Details on the driving simulator setup can be found in [34].

### Experimental design & procedure

The participants’ task was to drive safely and to maintain a target velocity of 120 kph while driving through a two-lane highway scenario. Participants completed three sessions in total, where one session took approximately nine minutes. The data analyzed here were recorded as part of a larger study that aimed at evaluating a tactile driving assistance system. We used only data recorded during a final baseline drive without the assistive device, as we were not interested in the effect of the assistance system on eye movements. Participants had already completed two drives before this second baseline, hence, we considered participants as being well trained on the task at this stage.

In order to reach the task goal of maintaining a speed of 120 kph, the experimental design of the scenarios made it necessary to overtake cars in front from time to time. This was realized by having the car in front of the ego car slowing down significantly. In total, twelve overtaking events were attained, which were considered here as trials. The onset of each trial was defined as the moment, where the front car started to slow down. The offset of each trial was defined as the moment where the ego car completed an overtaking maneuver by passing the white lane marks from the left lane back to the right lane. The inter-trial interval was varied to ensure that the onset of the situation was not predictable for the participants. Each trial or overtaking maneuver was split into two periods, one from the beginning of the trial until the change from the right onto the left lane (*before lane change*), and the second from the lane change until the end of the trial, indicated by a change back to the right lane (*after lane change*). Before the start of the actual experiment, all participants took part in several familiarization procedures (for details see [34, 35]).

Trials were additionally varied in task demand: Half of the trials were defined as *difficult* whereas the other half of the trials were defined as *easy*. Easy and difficult trials appeared in randomized order. The level of difficulty was varied based on the time left for the ego car to initialize a lane change once the car in front started to break. In the difficult trials this time was reduced to approximate the minimum that would still allow a maneuver without creating a crash with cars on the ego or left lane. In the easy trials, time left to start the lane change maneuver was chosen to allow for a relaxed action. Subjective data from questionnaires after the experiment as well as objective driving data indicated that participants did perceive differences in task difficulty throughout the experiment (see [34] for details).

In case participants did not manage to complete the overtaking scenario appropriately, i.e. created a crash or the scenario did not develop correctly due to deviations from the target velocity, trials were considered as invalid and did not enter any further analysis. Over the eleven subjects entering further analysis, the mean number of fixations in each scan path was 865.70 ± 74.21 (1 SD).

### Preprocessing

We first preprocessed the eye tracking recordings by mapping gaze points to eight AOI, defined based on their relevance to the task (Fig 1A. Fixations were computed in Pupil Labs using their default parameters. Fixations below 100 ms and above 1500 ms were excluded from the data analysis, as well as data points with a confidence value below 0.9, where confidence constitutes an assessment by the pupil detector on how reliably the pupil was tracked by the eye tracker at this point in time (Pupil Labs GmbH, see [36]).

### Information-theoretic data analysis

To quantify the predictability of gaze behavior, we estimated local variants of gaze transition entropy (GTE) as well as active information storage (AIS) from the scan path. In the following, we will formally introduce the two measures (see also [14]). To this end, we assume that a scan path is an observed time series **x** = (*x*_1_, *x*_2_, …, *x*_*i*_, …, *x*_*N*_) that consists of realizations of individual random variables, **X** = (*X*_1_, *X*_2_, …, *X*_*i*_, …, *X*_*N*_), indexed by time *i* ∈ {1, …, *N*} and with possible outcomes $x_{i}\in A_{X_{i}}=\{1,2,\ldots ,8\}$. We write *p*(*x*_*i*_) as a shorthand for *p*(*x*_*i*_ = *X*_*i*_), and *p*(*x*_*i*_|*x*_*i*−1_) as a shorthand for *p*(*X*_*i*_ = *x*_*i*_|*X*_*i*−1_ = *x*_*t*−*i*_).

#### Gaze transition entropy (GTE)

The GTE measures the remaining uncertainty about the location of a fixation, given knowledge of the immediate past fixation [10, 11]. GTE is calculated as the conditional entropy of the current fixation, *X*_*t*_, given the previous fixation, $X_{t-1}^{-}$,

$$\begin{array}{c} \begin{array}{cc} H(X_{t}|X_{t-1}) & =H\left(X_{t},X_{t-1}\right)-H\left(X_{t-1}\right)\\ & =-\sum_{X_{t-1}}p\left(X_{t-1}\right)\sum_{X_{t}}p\left(X_{t}|X_{t-1}\right)\log p\left(X_{t}|X_{t-1}\right)\\ & =-\sum_{X_{t},X_{t-1}}p\left(X_{t},X_{t-1}\right)\log p\left(X_{t}|X_{t-1}\right). \end{array} \end{array}$$ (1)

The GTE is upper-bound by the joint entropy, *H*(*X*_*t*_|*X*_*t*−1_) ≤ *H*(*X*_*t*_, *X*_*t*−1_), as well as the entropy of the next fixation, *H*(*X*_*t*_|*X*_*t*−1_) ≤ *H*(*X*_*t*_).

Note that the GTE measures the remaining uncertainty in the next fixation, *X*_*t*_ given the *immediate past* fixation, *X*_*t*−1_, only. Hence, it does not take into account information that fixations further in the past, *X*_*t*−*l*_, where *l* > 1, provide about *X*_*t*_.

#### Active Information Storage (AIS)

The AIS [15] quantifies how much information a processes’ past state, $X_{t-1}^{-}$, contains or stores about its next value, *X*_*t*_, and may be interpreted as the predictability of *X*_*t*_ from its immediate past [15, 37]. AIS is calculated as the mutual information (MI) between *X*_*t*_ and $X_{t-1}^{-}$,

$$\begin{array}{c} \begin{array}{cc} AIS(X_{t}) & =I(X_{t-1}^{-};X_{t})\\ & =H\left(X_{t}\right)-H\left(X_{t}|X_{t-1}^{-}\right)=H\left(X_{t-1}^{-}\right)-H\left(X_{t-1}^{-}|X_{t}\right)\\ & =H\left(X_{t},X_{t-1}^{-}\right)-H\left(X_{t}|X_{t-1}^{-}\right)-H\left(X_{t-1}^{-}|X_{t}\right)\\ & =\sum_{x_{t},x_{t-1}^{-}}p\left(x_{t},x_{t-1}^{-}\right)\log\frac{p(x_{t}|x_{t-1}^{-})}{p(x_{t})}. \end{array} \end{array}$$ (2)

Here, the past state $X_{t-1}^{-}$ is defined as a collection of random variables, $X_{t-1}^{-}$ selected from a set of past variables $\left\{X_{t-1},\ldots ,X_{t-t_{l}},\ldots ,X_{t-l_{max}}\right\}$ (see next section).

The AIS is low for highly unpredictable processes or processes with low entropy. Processes with low entropy visit few processes with high probability, i.e., contain little information to be predicted. On the other hand, the AIS is high for processes that are highly regular or predictable and have high entropy, i.e., visit many different states with equal probability [15, 37]. AIS is lower-bound by zero (for processes that store no information), 0 ≤ *AIS*(*X*_*t*_), and it is upper-bound by the entropy of either of the two variables involved, $AIS\left(X_{t}\right)\leq H\left(X_{t}\right),H\left(X_{t-1}^{-}\right)$, as well as by the joint entropy $AIS\left(X_{t}\right)\leq H\left(X_{t},X_{t-1}^{-}\right)$ (Eq 2).

**Fig 2:**
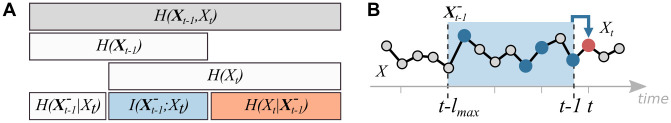
Schematic illustration of AIS estimation and relationship with other information-theoretic measures. (A) Schematic illustration of relationship between AIS (blue box), conditional entropy of the next fixation given the past state (orange box) and the joint entropy between past state and next fixation (grey box). For $X_{t-1}^{-}=X_{t-1}$, the conditional entropy is equivalent to the GTE. (Adapted from [38]). (B) Schematic illustration of the non-uniform embedding (modified from [14]). The blue box indicates all past variables up to a maximum lag, *l*_*max*_, that are considered during the optimization of the past state for AIS estimation. The red marker indicates the next fixation, *X*_*t*_, and blue markers indicate the variables selected by the optimization that comprise the optimized past state, $X_{t-1}^{-}$.

The AIS is related to GTE by the entropy of the next fixation and the joint entropy of the next and past fixation (Fig 2A). In cases, where the relevant past is defined or identified to comprise only a single past fixation, i.e., $X_{t-1}^{-}=X_{t}$, the AIS and GTE are complementary such that they sum up to the entropy of *X*_*t*_,

$$\begin{array}{c} H\left(X_{t}\right)=GTE\left(X_{t}\right)+AIS\left(X_{t}\right)=H\left(X_{t}|X_{t-1}\right)+I\left(X_{t},X_{t-1}\right). \end{array}$$ (3)

Accordingly, both measures are complementary in their interpretation—while the GTE measures the remaining uncertainty in the next fixation (Eq 1), the AIS (Eq 2) provides a more direct measure of predictability as it quantifies how much uncertainty in the next fixation can be “resolved” from the fixation’s immediate past [37].

#### Local interpretation of information-theoretic measures

Information-theoretic quantities, such as the entropy or MI, are measures that describe average properties of random variables [38]. However, it has been noted that for these average quantities local counterparts exist [39, 40], which estimate information-theoretic measures also for individual realizations of random variables. In practice, local information-theoretic measures thus allow to quantify the predictability or (conditional) information content of individual samples, e.g., individual fixations in a scan path. Examples for the application of local or point-wise information-theoretic measures can be found in natural language processing (e.g., [41]), in complex systems analysis [31, 42], biology [33, 43], or robotics [44].

The local variant of the AIS, the local active information storage (LAIS) [15], is obtained by dropping the summation in Eq 2 and describes the information that a specific realization of the past state, $x_{t-1}^{-}$, provides about a specific next state, *x*_*t*_,

$$\begin{array}{c} LAIS\left(x_{t}\right)=i\left(x_{t-1}^{-};x_{t}\right)=\log\frac{p(x_{t}|x_{t-1}^{-})}{p(x_{t})}, \end{array}$$ (4)

where $x_{t-1}^{-}$ and *x*_*t*_ are realizations of $X_{t-1}^{-}$ and *X*_*t*_. The lower-case *i* denotes the local MI. Hence, the AIS is obtained as the expected value of the LAIS over all realizations (*x*_*t*_, **x**_*t*−1_) (Eq 4), or it can be calculated as the average LAIS over all samples observed [45],

$$\begin{array}{c} AIS\left(X_{t}\right)={\left\langle LAIS\left(X_{t}\right)\right\rangle }_{i}. \end{array}$$ (5)

Other than the AIS, the LAIS can take on negative values whenever $p\left(x_{t}|x_{t-1}^{-}\right)<p\left(x_{t}\right)$, i.e., the probability of *x*_*t*_ occurring given $x_{t-1}^{-}$ occurs, is lower than the a-priori probability of *x*_*t*_ occurring. Vice versa, if the probability of *x*_*t*_ occurring given $x_{t-1}^{-}$ occurs is greater than the a-priori probability of *x*_*t*_ occurring, i.e., $p\left(x_{t}|x_{t-1}^{-}\right)>p\left(x_{t}\right)$, the LAIS becomes positive. Intuitively, local AIS may be interpreted as individual values providing information about the occurrence of the next value (positive LAIS), or as individual values being *misinformative* about the next value. Note, however, that the LAIS always averages to a positive quantity.

Similarly, we can define the local conditional entropy [45] and similarly a local gaze transition entropy (LGTE) as

$$\begin{array}{c} LGTE\left(X_{t}\right)=h\left(x_{t}|x_{t-1}\right)=-\log p\left(x_{t}|x_{t-1}\right). \end{array}$$ (6)

This local conditional entropy describes the information content of realization *x*_*t*_, given *x*_*t*−1_ is observed. The local conditional entropy, similarly to its average, follows the chain rule,

$$\begin{array}{c} h\left(x_{t}|x_{t-1}\right)=h\left(x_{t},x_{t-1}\right)-h\left(x_{t}\right), \end{array}$$ (7)

and for an assumed past state $X_{t-1}^{-}=X_{t-1}$, LGTE and LAIS sum to the local entropy or information content of *x*_*t*_,

$$\begin{array}{c} h\left(x_{t}\right)=LGTE\left(x_{t}\right)+LAIS\left(x_{t}\right)=h\left(x_{t}|x_{t-1}\right)+i\left(x_{t-1}^{-};x_{t}\right). \end{array}$$ (8)

A last important point when considering local information measures is that even though these measures describe information-theoretic properties of *individual* samples, their estimation still requires to include *all* available samples. More formally, local measures are obtained by evaluating probability distributions, *p*(⋅), used in calculating LGTE and LAIS (Eqs 4 and 6), for individual realizations, *x*_*t*_. These probability distributions, *p*(⋅), still have to be estimated from all available data points. Hence, estimates are local in a sense that they provide the information content or information contribution of an individual realizations, but they are global as they require the consideration of *all* available observations to arrive at an estimate of *p*(⋅).

#### Estimating LAIS from scan path data

Formally, AIS is defined as the MI between the next state of a process and its whole semi-infinite past [15]. To apply and estimate AIS in practice, the semi-infinite past is replaced by a past state, $X_{t-1}^{-}$, that is designed to contain all relevant past information about *X*_*t*_, such that *X*_*t*_ is conditionally independent of all earlier observations given $X_{t-1}^{-}$ [15, 46, 47]. This approach assumes that the process can be modelled as a higher-order Markov chain. Different approaches for constructing such a past state have been proposed, where we here used a so-called non-uniform embedding for time series [47, 48], with an algorithmic realization proposed in [29, 30].

Formally, we define $X_{t-1}^{-}$ such that $p\left(X_{t}|X_{t-1},X_{t-2},\ldots ,X_{0}\right)=p\left(X_{t}|X_{t-1}^{-}\right)$, i.e., the next value, *X*_*t*_, is conditionally independent of all past variables, *X*_*t*−*l*_, *l* > *l*_*max*_, given $X_{t-1}^{-}$. Here, *l*_*max*_ is the maximum lag in $X_{t-1}^{-}$. A non-optimal choice for $X_{t-1}^{-}$ may lead either to underestimation of AIS if relevant information is not covered by $X_{t-1}^{-}$. Conversely, if non-relevant variables are included in $X_{t-1}^{-}$, the AIS may be artificially inflated due to an increasingly large and thus potentially undersampled past state.

To optimize $X_{t-1}^{-}$, we applied a recently proposed algorithm [29, 30] that uses a greedy forward-selection approach to optimize the non-uniform embedding. The algorithm iteratively includes past variables if they provide significant, additional information about *X*_*t*_, conditional on all already selected variables (see also [30]). In other words, a past variable is included if its MI with *X*_*t*_, conditional on all already selected variables, is significant. The algorithm terminates if no variable provides any new information about *X*_*t*_, hence providing an automatic stopping criterion for constructing the past state.

The optimized embedding,

$$\begin{array}{c} X_{t-1}^{-}=\{X_{t-l}\},\;l\in [1,l_{max}], \end{array}$$ (9)

contains all relevant information stored in the past of *X*_*t*_, while being as small as possible such as not to lead to an under-sampling of the past state. Here, variables up to a maximum lag *l*_*max*_ are tested for inclusion, where *l*_*max*_ has to be provided by the user. For optimization of the past state and estimation of LAIS and LGTE we used the IDTxl Python toolbox [29] with estimators from the JIDT toolbox as backend [49].

All information-theoretic measures are estimated from discrete scan-path data using plug-in estimators [50]. These estimators that are known to exhibit a bias due to finite sampling (e.g., [51–53]). Hence, we use non-parametric permutation testing to test MI-estimates and conditional MI-estimates for statistical significance [54]. In particular, the AIS estimation algorithm applied in the present work uses a hierarchical permutation testing scheme to handle estimator bias, while controlling the family-wise error rate during repeated testing [30]. This test allows to determine whether past fixations carry information about future fixations at all.

### Statistical testing using linear mixed models

To test for changes in gaze behavior related to changes in the driving task, as well as based on trial-difficulty, we fitted a linear mixed-effects model (LMEM) [55] to normalized LAIS and LGTE estimates, that were averaged per participant and trial period. We tested for an effect of the factor *difficulty* with levels *easy* and *hard*, and for an effect of factor *trial period* with levels *baseline*, *before lane change*, and *after lane change*, where the second two periods comprise the actual task (see also Fig 1B). We further tested for an interaction of both factors and added *participant* as a random effect. We allowed for a variation of the intercept and the slope for each participant.

We encoded factor *trial period* using a Helmert coding scheme with a comparison of *baseline* vs *before lane change*, and a comparison of these two levels with *after lane change*, in order to detect changes in scan pattern predictability in comparison to previous trial intervals. We encoded factor *difficulty* using deviation coding, comparing the first factor level, *easy*, with the grand mean.

We used the lme4-package [56] written in R for model fitting. In general, testing for the significance of a fixed effect in LMEMs is not straightforward because it is not clear what the denominator degrees of freedom for obtaining the *p*-values are. A common approach to evaluate the statistical significance of fixed effects are likelihood-ratio test (e.g., [57]), however, this test was reported to be anti-conservative for smaller sample sizes [58]. Hence, we here followed the recommendation by Luke et al. [58] and for calculation of *p*-values used Satterthwaite’s approximation of the degrees of freedom [59] as implemented in the lmerTest R-package [60].

## Results

### Fixation statistics

Before analyzing gaze patterns using information-theoretic methods, we investigated whether our experimental manipulation had an effect on the driver’s viewing behavior by investigating basic eye movement statistics and their relationship with the experimental manipulations. If the requirements of the driving task affected the driver’s viewing strategy, we would expect a change in fixation measures according to the changes in the *trial period* and *difficulty*.

#### Proportion of fixated AOI

**Fig 3:**
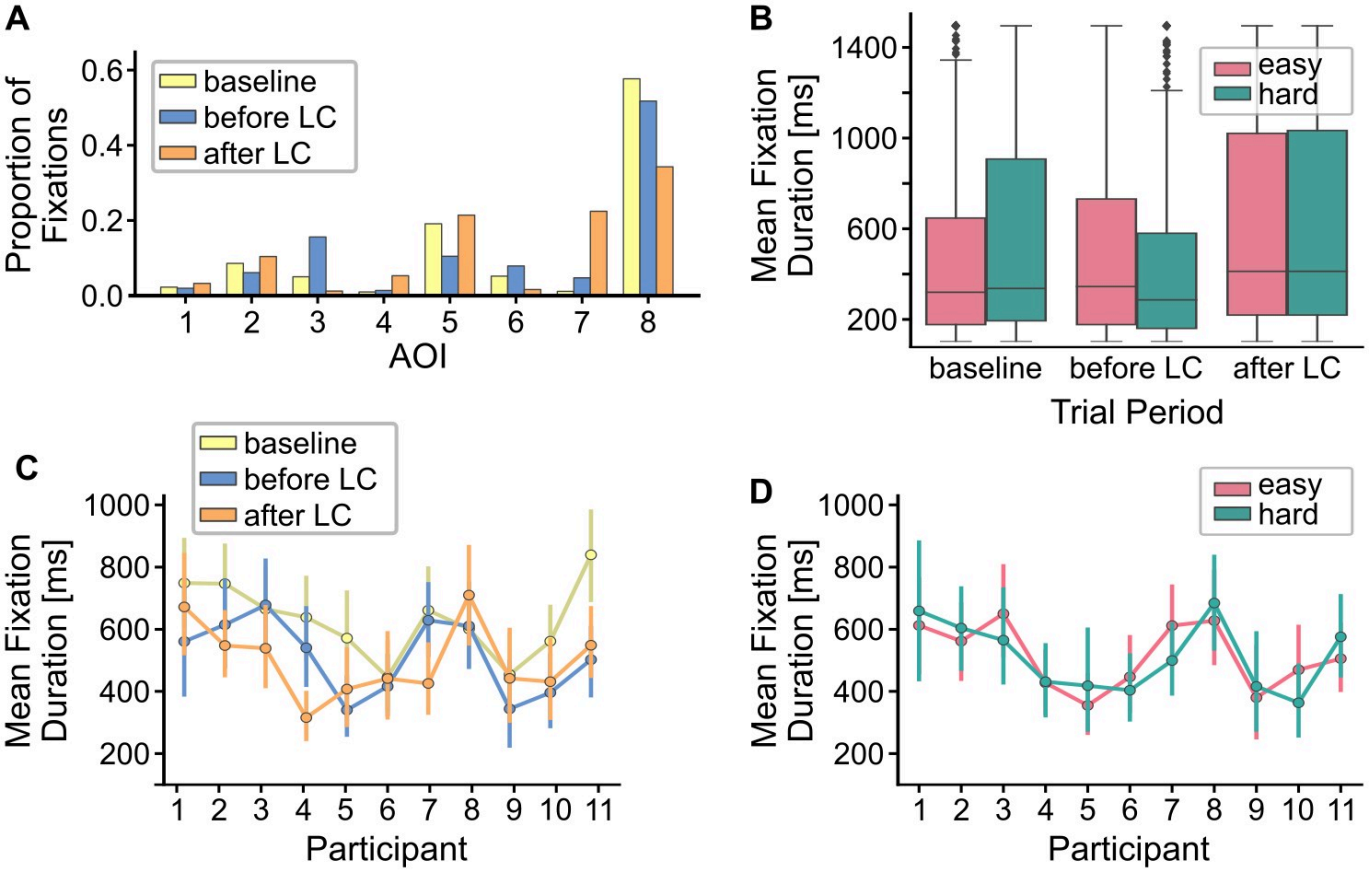
Proportion of fixations and mean fixation duration. (A) Relative fixation proportions for each AOI across all participants in the three different trial periods (LC = lane change). (B) Distribution of mean fixation duration across participants for the three trial periods and two levels of difficulty including (whiskers indicate 95% confidence intervals). (C) Mean fixation duration per participant for each *trial period* (error bars indicate 95% confidence intervals). (D) Mean fixation duration per participant for both *difficulty* levels (error bars indicate 95% confidence intervals).

First, we inspected the relative proportion of fixated AOI in the different trial periods. Fig 3A shows the relative proportion of fixations across all participants in the three trial periods (*baseline*, *before lane change*, and *after lane change*). As expected, by far most of the fixations landed within AOI 8 (*ego lane*, Fig 1A), for all three trial periods. Nevertheless we would expect to observe a higher proportion of fixations on the rear mirrors and neighboring lanes during the preparation and overtaking procedure compared to the *baseline* period. This was indeed the case. The highest proportion of fixations on the left rear mirror was observed in the *before lane change* period, while the highest proportion of fixations on the right rear mirror was observed in the *after lane change* period.

#### Mean fixation duration

In a next step, we tested mean fixation duration for the three trial periods and the two levels of difficulty (Fig 3B). We ran a 2x3 repeated measures ANOVA with factors *difficulty* (*easy* versus *hard*) and trial period (*baseline* versus *before lane change* versus *after lane change*). On average, mean fixation duration were longest in the *baseline period* (*m* = 629.92 ms, *SEM* = 19.84 ms) compared to fixations during the trial, i.e., *before lane change* (*m* = 530.59 ms, *SEM* = 20.33 ms) and *after lane change* (*m* = 501.48 ms*s*, *SEM* = 20.03 ms). We observed a significant main effect for the factor *task period* (*F*(2) = 7.515, *p* < 0.01). Post-hoc paired-sample t-tests revealed significant differences between the *baseline* period and both task periods (*after lane change* (*T* = 2.59, *p* = 0.02) and *before lane change* (*T* = −2.29, *p* = 0.03), uncorrected).

The manipulation of trial difficulty had only minor effects on the average fixation duration (*easy*: *m* = 560.77 ms, *SEM* = 16.00 ms, *hard*: *mean* = 558.58 ms, *SEM* = 17.20 ms). Accordingly, we did not find an effect of *difficulty* on mean fixation duration (*F*(1) = 0.242, *p* = 0.63), nor did we find a significant interaction between *difficulty* and *trial period* (*F*(2) = 2.000, *p* = 0.16).

The results indicate that on average participants showed longer fixation duration in the *baseline* period than during the overtaking maneuver, although differences were only marginally significant after Bonferroni correction. Somewhat surprisingly our manipulation of *difficulty* did not affect fixation duration. This stands in contrast to earlier results based on the driving data [35] where we found that trial difficulty had a significant effect on the driving performance, indicating that our experimental manipulation was still valid.

#### Robustness of inter-individual differences

Lastly, we analyzed the robustness of inter-individual differences in mean fixation duration. To that end, we computed the spearman correlation coefficients between the different experimental conditions across participants. Such an approach has been previously suggested by Andrews et al. [61].

Mean fixation durations varied quite substantially between participants across all experimental conditions (range: 427 ms to 682 ms). Fig 3C and 3D show the mean fixation duration for the different *trial periods* and the two levels of *difficulty* as a function of participants. We found a significant correlation between easy and hard trials across participants (*r* = 0.7, *p* < 0.05), indicating that the inter-individual differences in mean fixation duration were consistent across these two conditions. We did not observe significant correlations across participants between the task periods. This suggests that variations in mean fixation duration in these conditions were differently affected by the experimental manipulation for individual participants, resulting in less consistent inter-individual differences in mean fixation duration.

Taken together, results on basic fixations statistics indicate that our experimental manipulations had an effect on the participant’s eye movement behavior. We find differences in mean fixation duration as well as on the overall dispersion of gaze depending on the driving task. This suggests that our data are valid for further investigation.

### Information-theoretic data analysis

After analyzing basic properties of gaze behavior, we proceeded to analyze the regularity of scan paths using information-theoretic methods. In particular, we compared the effect of both *trial period* as well as *difficulty* on the regularity of scan paths as measured by both the GTE and AIS.

#### Optimized past states

For the estimation of AIS, we first identified relevant past fixations by applying an estimation algorithm proposed in [29, 30]. We optimized the past states individually for each participant while allowing the optimization procedure to test for dependencies up to a maximum lag, *l*_*max*_, of 5 previous fixations. This means that the optimal past state (optimal number of lags) was estimated for each participant individually in a data-driven fashion.

Through application of the estimation algorithm proposed in [29, 30] we tested hypotheses H1 and H2, raised in the introduction: first, the estimation algorithm optimizes the past state used for AIS estimation by identifying all past variables of a time series that jointly provide information about the time series’ next state. Thus, the optimization returns whether only a single previous fixation provides information about the next fixation or whether fixations with larger time-lags are predictive of the next fixation. Hence, the algorithm directly tests the first hypothesis that scan paths can not generally be modeled by a first-order Markov chain. Second, the algorithm optimizes the past state in a data-driven fashion and can be applied individually for each participant. We thus tested our second hypotheses, which states that individuals may differ in the time horizon of past fixations being predictive for future gazing behavior.

**Fig 4:**
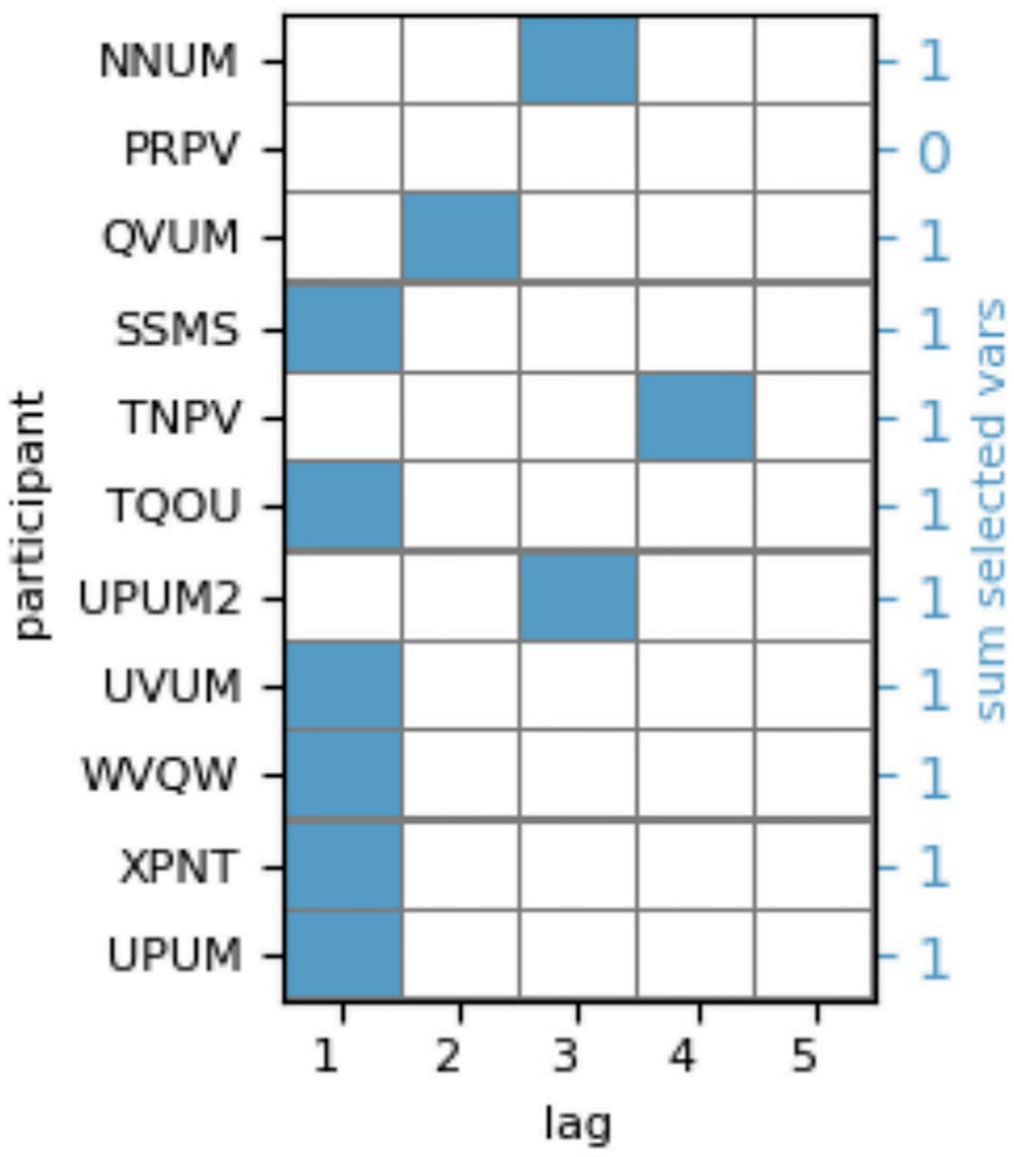
Selected past variables per participant. Lags, *l*, of past variables used for AIS estimation as identified by the estimation algorithm for each participant.

In accordance with our first two hypotheses, the optimization returned a wide variety of selected past variables over participants, where in 4 out of 11 participants, variables with lags greater one, *l* > 1, were selected and for one participant no significant AIS was found (Fig 4). For participants with lags *l* > 1, fixations prior to the last fixation provided significant information about the next fixation and were relevant for quantifying the predictability of the scan path. The result provides evidence that indeed the first-order Markov chain assumption does not hold per se for each individual (H1). Furthermore, the variability in lags, *l*, provides evidence for an inter-individual variance in viewing behavior, in particular the time horizon over which fixation sequences are predictive of future fixations (H2).

#### Definition of experimental groups based on identified long-range dependencies

In a next step, we tested our third hypothesis that an individually optimized scan path representation, which takes into account long-range dependencies in viewing behavior, should result in a better distinctiveness of user states. To this end, we split participants into two groups based on whether past state optimization, identified a significant information contribution of the single past fixation only (*l* = 1), or whether the optimization returned a significant contribution of variables with a greater lag (*l* > 1) (one participant was excluded from further analysis as no significant relationship between past and next fixation had been found.).

For participants in both groups, we estimated both the GTE and AIS from individual scan path data and analyzed whether predictability of gaze behavior changed as a function of *difficulty* (*easy*, *hard*) and *trial period* (*baseline*, *before lane change*, *after lane change*). To make optimal use of the available data, i.e., consider the maximum amount of data for estimation of information-theoretic measures, we estimated the local variants of both measures, LGTE and LAIS, from the complete consecutive scan path over the whole recording interval. To analyze changes in scan path predictability during the trial periods within the recording, we performed statistical tests on averaged local estimates within these periods. We thus tested whether the predictability of fixations differed between the baseline interval and the overtaking maneuver, and whether predictability varied between easy and hard trials.

#### Changes in information-theoretic measures as a function of trial period

**Fig 5:**
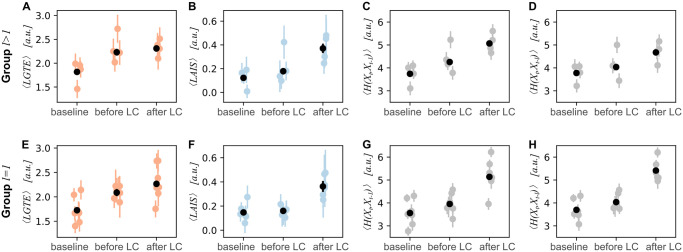
Mean values of LGTE (orange), LAIS (blue), and joint entropy (gray) as a function of trial period. Mean per trial period for participants with lag *l* > 1 for (A) LGTE, (B) LAIS, (C) joint entropy between next fixation and past fixation at *t* − 1 (fixations used for LGTE estimation), (D) joint entropy between next fixation and past fixation at *t* − *l* (fixations used for LAIS estimation). Mean per trial period for participants with lag *l* = 1 for (E) LGTE, (F) LAIS, (G) joint entropy between next fixation and past fixation at *t* − 1 (fixations used for LGTE estimation), (H) joint entropy between next fixation and past fixation at *t* − *l* (fixations used for LAIS estimation). Error bars indicate the standard error of the mean.

For both groups with optimized past states *l* > 1 and *l* = 1, we estimated LAIS as well as LGTE (Fig 5A, 5B, 5E and 5F). Note that both measures increased during the task periods compared to the baseline, indicating a decrease in predictability for the GTE (more uncertainty in the next fixation) and an increase in predictability for LAIS (more information in the next fixation is predictable from its past).

Note that changes in predictability as measured by raw LAIS and LGTE were not complementary. However, complementary results can only be expected given constant entropy of the involved variables in the compared conditions. Hence, an increase in both LAIS *and* GTE between baseline and task interval, especially for the *l* = 1 group where Eq 3 applies directly, indicates a higher entropy in the task than in the baseline, leading to a higher overall information content to be predicted in the next fixation (see also Fig 2A). Indeed, we found such an increase in the joint entropy of both the next fixation at time *t* and the respective past fixation at either *t* − 1 or *t* − *l* (Fig 5C, 5D, 5G and 5H). Hence, for further analysis, we normalized the mean LAIS and mean LGTE by the mean joint entropy for the respective *trial period* and participant.

#### Effects of trial difficulty and trial period on normalized information-theoretic measures

To test for effects of both *difficulty* and *trial period* on the predictability of viewing behavior, we fitted LMEMs [55] separately to both normalized mean LAIS and normalized mean LGTE, as well as both groups, *l* > 1 and *l* = 1 (Tables 1 and 2).

**Table 1:** Linear mixed-effect model results for normalized *LAIS* and *LGTE* estimates for participant group with past state *l* > 1.

Measure	Predictor	Estimate	*SE*	$\hat{df}$	*t*	*p*
〈*LGTE*〉/〈*H*〉	*difficulty*	-0.0011	0.0082	120.0	-0.139	0.8900
	*trial period:BLC*	0.0583	0.0366	3.2	1.593	0.2050
	*trial period:ALC*	-0.0757	0.0369	3.8	-2.051	0.1130
〈*LAIS*〉/〈*H*〉	*difficulty*	0.0019	0.0104	3.3	0.186	0.8634
	*trial period:BLC*	-0.0028	0.0189	72.3	-0.150	0.8812
	*trial period:ALC*	0.0620	0.0250	36.9	2.481	0.0178*

* *p* < 0.05;

**p < 0.01;

***p < 0.001.

**Table 2:** Linear mixed-effect model results for mean normalized *LAIS* and *LGTE* estimates for participant group with past state *l* = 1.

Measure	Predictor	Estimate	*SE*	$\hat{df}$	*t*	*p*
〈*LGTE*〉/〈*H*〉	*difficulty*	-0.0053	0.0072	194.1	-0.736	0.4628
	*trial period:BLC*	0.0057	0.0212	6.3	2.673	0.0354*
	*trial period:ALC*	-0.0093	0.0279	6.0	-3.321	0.0160*
〈*LAIS*〉/〈*H*〉	*difficulty*	-0.0036	0.0061	190.0	-0.594	0.5530
	*trial period:BLC*	-0.0092	0.0141	190.0	-0.649	0.5174
	*trial period:ALC*	0.0380	0.0182	190.0	2.085	0.0384*

* *p* < 0.05;

**p < 0.01;

***p < 0.001.

For the *l* > 1 group we expected potential differences in the effects of *trial period* and *difficulty* on normalized LAIS compared to normalized LGTE. In particular, in this group, the first-order Markov chain assumption was violated. Therefore, LGTE estimates should presumably reflect changes in predictability less accurately than LAIS estimates. The LAIS was estimated based on the individually optimized past state beyond a single past fixation, while the LGTE, by definition, was estimated only considering the last (presumably non-optimal) fixation. Hence, the LGTE may underestimate the true predictability leading to a potentially worse identification of changes in gaze behavior.

**Fig 6:**
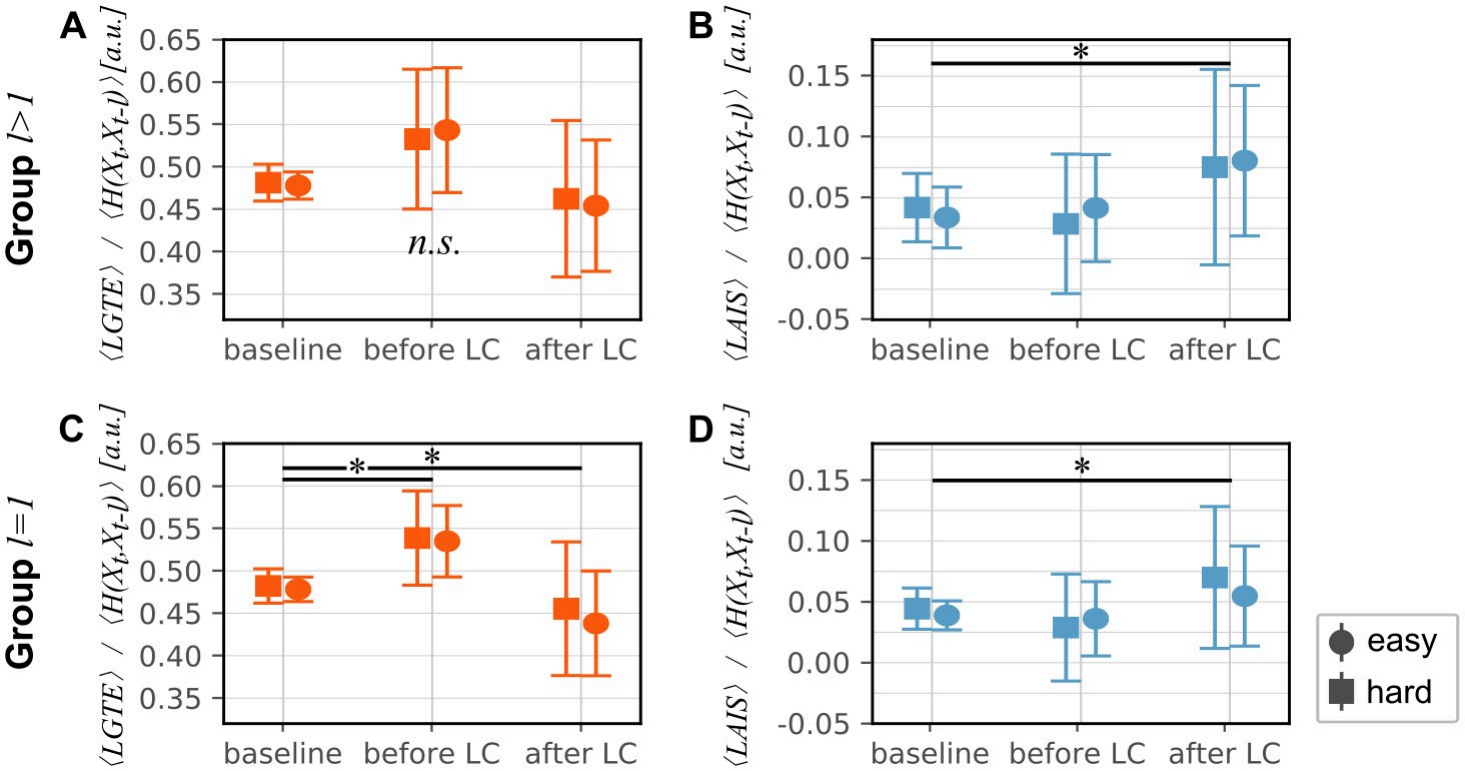
Predicted values of mean LGTE (orange) and mean LAIS (blue) by linear mixed-effects model (LMEM). **Error bars indicate confidence intervals**. (A) Mean LGTE values normalized by mean joint entropy for *l* > 1, (B) mean LAIS values normalized by mean joint entropy for *l* > 1, (C) mean LGTE values normalized by mean joint entropy for *l* = 1, (D) mean LAIS values normalized by mean joint entropy for *l* = 1.

Indeed, for group *l* > 1 we did not find a significant effect of *trial period* on normalized LGTE (Fig 6A), while we found a significant decrease in the normalized LAIS in the *after lane change* interval compared to the previous intervals (*t*(36.9) = 2.481, *p* = 0.0178*, Fig 6B).

For the *l* = 1 group, on the other hand, we expected a similar outcome of the analysis for both measures, as for this group both LAIS and LGTE are estimated only considering a single past fixation (here, the first-order Markov chain assumption was not violated). Here, we found differences for both measures between the *after lane change* interval compared to the previous intervals, normalized LGTE decreased significantly (*t*(6.0) = −3.321, *p* < 0.05, Fig 6C) and normalized LAIS increased significantly (*t*(190) = 2.085, *p* < 0.05, Fig 6D). Both results indicate an increased predictability of viewing behavior in the second task interval. Also LGTE increased in the *before lane change* interval compared to the baseline (*t*(6.3) = 2.673, *p* < 0.05), indicating a decrease in predictability in the first task interval.

We did not find an effect of *difficulty* on either normalized LAIS or LGTE in any of the groups. Hence, results on information-theoretic analysis of viewing behavior were consistent with results on basic viewing statistics.

In sum, results are in line with our third hypothesis and provide evidence that accounting for an individually optimized past state is beneficial for detecting changes in viewing behavior. Furthermore, normalizing mean LGTE and LAIS by the joint entropy led to the expected complementary behavior of both measures. Also, normalizing led to qualitative changes in the effect of *trial period* on LGTE (overall decrease in normalized versus an increase in raw LGTE), highlighting the importance of considering the total information present in the scan path when evaluating relative changes in predictability of gaze behavior.

## Discussion

The present work investigated the role of long-term dependencies between fixations in estimating the predictability of scan paths during a dynamic driving task. We proposed to quantify predictability in fixation sequences using active information storage (AIS), which is able to account for more long-range influences between fixations. We demonstrated that the proposed approach is able to detect changes in viewing behavior more reliably compared to the commonly used gaze transition entropy (GTE). In particular, we found evidence supporting three hypotheses raised in the beginning: 1) we showed that in a dynamic task the assumption that scan path data are sufficiently modeled by first-order Markov chains does not hold in general, 2) the time horizon of past fixations being predictive for the next fixation varies between participants, and 3) accounting for detected long-range dependencies between fixations in a personalized way is beneficial for the meaningful quantification of the predictability of scan paths.

### Using active information storage to quantify scan path predictability

We proposed to quantify predictability in scan path by estimating the active information storage (AIS) [15] using a recently proposed estimation algorithm [29, 30]. The algorithm optimizes the past fixations used for the estimation of AIS in a data driven fashion, and is thus able to account for potential long-range dependencies in a personalized fashion.

By applying this approach to data recorded during a driving simulator experiment, we showed that for a subset of the participants, fixations were predictive of future gaze behavior with a delay of up to four fixations. These findings are in line with our earlier results applying AIS to scan path data in a static viewing task [14]. We further showed that for these participants (*l* > 1), an increase in the predictability of gaze behavior during an overtaking maneuver could only be detected in the AIS estimate while the gaze transition entropy (GTE) failed to uncover these changes. In other words, changes in gaze behavior could only be detected when discarding the first-order Markov assumption and accounting for the participants’ individual longer time horizon over which fixations were predictive of future gaze behavior. As hypothesized, attempting to quantify the scan path predictability using GTE while the first-order Markov assumption was violated, led to an underestimation of scan path predictability during the task. For participants for which the optimization approach supported the assumption of a first-order Markov chain (*l* = 1), both the LAIS and GTE were able to identify a significant increase in predictability of gaze behavior, indicating that both measures arrived at converging results if the first-order Markov assumption was met.

We want to highlight that in cases where the assumption of scan paths following a first-order Markov chain fails, the GTE does not measure the total predictability of a scan path from its past. Here, the AIS is a direct measure of the concept of predictability [15, 37, 62], in particular when optimizing the past state used for its calculation such as to include all relevant past information. While GTE has proven to be a valuable marker of gaze behavior in a variety of tasks (e.g., [11]), it may miss long-range temporal relationships in scanning behavior, in particular during dynamic tasks such as driving, which require more planning by the observer.

In sum, we provide evidence that more long-range temporal relationships between fixations should be accounted for in a personalized fashion when quantifying the predictability of gaze behavior. This finding is in line with earlier findings using non-information-theoretic approaches to describing scan path data (e.g., [12, 63], see also [14]). We here furthermore presented a flexible approach for estimating predictability in scan path data using AIS, which handles long-range dependencies as well as their inter-individual variation.

### Relationship between predictability and scan path entropy

We found that the joint entropy of next and past fixation increased during the task compared to the baseline driving. The increase in entropy means that participants fixated more AOI with equal probability such that the visual space was more thoroughly explored. This result is in line with our findings on proportions of fixations, where we found that almost 80% of the fixations during baseline driving were in AOI 5 (*dashboard*) or AOI 8 (*ego lane*), while this proportion was lower for the task intervals.

We normalized AIS and GTE by the joint entropy to exclude the possibility that changes in AIS and GTE were solely due to a corresponding change in the scan path’s stationary entropy. In the latter case, changes in raw AIS and GTE would reflect a change in the overall richness of the visual scanning behavior, i.e., the total information to be predicted, but may not be due to an actual change in the predictability of the scanning behavior. Hence, we want to point out the importance of controlling for the overall predictability of the scan path under analysis (e.g., [37]), which is rarely done in the analysis of scan path predictability [11].

Indeed, we found an increase in predictability after normalizing GTE and AIS. Together with the observed increase in joint entropy, these results indicate that gaze behavior became both more dispersed and more regular during the task. In other words, gaze patterns became richer in the overall information, but also followed a more regular trajectory through the visual space, which indicates that the participants’ exploration of the visual space branched less often and such that fixation sequences became more predictable.

### Increased scan path predictability and task demand

Previous studies that investigated GTE as a marker for changes in task demand or user state found mixed results. Gotardi et al. [64] showed that drivers showed more erratic viewing behavior and thus higher visual entropy under conditions that induced anxiety during driving. Similarly, Shiferaw et al. [5] for example found an increase in GTE in sleep deprived drivers. Moreover, increases in stationary entropy were found in surgeons under higher task load [20, 65]. These results indicate that the regularity of scanning behavior can decline under conditions in which observers feel under pressure or are suffering from context-driven cognitive impairments, thus resulting in higher entropy values. On the other hand, a number of studies report a decrease in GTE as a function of increasing task difficulty. Schieber et al. [66], for example, found a decrease in GTE during driving when participants had to solve a visuo-spatial task while driving. Similarly, decreases in GTE were demonstrated in a pattern-recognition task with increasing task-difficulty [67] and in fighter pilots in high complexity emergency situations [68]. These results indicate that viewing behavior can become more deterministic under conditions that require a high cognitive engagement, see [11] for a review. In line with existing results, we expected to observe changes in GTE and likewise in AIS associated with our experimental manipulations.

While driving, we observed an effect of the trial period on both fixation statistics and scan path predictability. The regularity of the gaze behavior increased in particular during the second overtaking period with respect to the previous trial periods. It could be argued that the change from the left back onto the right lane poses a more difficult task that may impose more stress, compared to previous trial intervals and therefore predictability should drop. For example, [69] suggests that driving on straight motorways is considered an easier driving task compared to winding or mountain roads. In the baseline condition, drivers were instructed to maintain constant speed while remaining on the right lane, which requires only attention to the vehicle in front of the ego car and a monitoring of the speed. The second trial period, *before lane change*, required monitoring of the left lane and the car in front on the ego car, while the second part of the overtaking maneuver comprised of monitoring oncoming traffic from behind, traffic in front, and identifying a gap on the right line while monitoring speed. Hence, the last trial period required increased attention of the driver to traffic on both lanes as well as the monitoring of the car that was overtaken. On the other hand, overtaking is also a highly trained task that may require more cognitive resources but presumably also involves a highly regular scheme of fixation sequences. In line with the results of [66–68], it seems therefore not surprising that we find a significant increase in scan path predictability during the overtaking maneuver compared to the baseline drive. It has been reported that in unknown routes, fixations are dispersed widely over the roadway with most fixations above and to the right of the road [70]. This might additionally explain the more irregular scanning behavior in the baseline period.

Somewhat surprisingly, for trial difficulty we did not find an effect on the regularity of gaze patterns. It could have been assumed that in the difficult trial condition, predictability of the scan paths would have dropped relative to the easy trial condition. We found an effect of trial difficulty on driving relevant parameters for this experiment in an earlier work, indicating that our experimental manipulation was generally valid ([35]). However, neither fixation statistics, nor our quantification of scan path predictability revealed an effect. One reason for this could be that participants were already highly trained on the task at this point of the experiment, which could have led to an overall more similar gaze behavior independent of the trial difficulty. Another potential reason is that both difficulty conditions can be generally classified as rather demanding. Even though we included the additional differentiation in *easy* and *hard* trials depending on the time left for initiating the overtaking maneuver without creating a crash, both scenarios can be regarded as high criticality situations. This might explain why we find a clear difference in the gaze behavior between baseline driving and the overtaking periods, but not between the two difficulty levels.

### Increased scan path predictability and top-down control

Shiferaw et al. [11] hypothesized that increased GTE is a result of an interaction between scene complexity and task demand and constitutes an estimate for top-down modulation of gaze control. More precisely, if scene complexity and task demand are high, more control is required. Furthermore, if complexity and demand are held constant and only observer state is manipulated, it is suggested that more engagement with the task, e.g., more top-down control is accompanied by higher GTE (lower predictability).

We find higher entropy, i.e., a greater exploration of the scene, which may result from a higher task demand that requires a more thorough visual exploration of the scene. However, opposed to the hypothesis formulated in [11], we do not find an increase in GTE, i.e., a lower predictability with highest task demand. Instead, the observed increase in predictability could hint at the execution of a high-level behavior plan necessary to realize the overtaking task. In fact, only recently it was compellingly illustrated in a visual search task that fixation sequences can be planned ahead [27]. In line with these results, it can be hypothesized that in a highly trained task like our overtaking scenario, drivers execute fixation planning leading to a higher predictability of scanning behavior.

However, based on the present experimental design we can not draw final conclusions on whether the observed increase in scan path predictability is a direct result of increased top-down influence. Scanning behavior is not only driven by top-down influences, but also by properties of the visual input, e.g., visual saliency, such that scan path predictability may even depend on the predictability of the location of salient objects. To disentangle both top-down and bottom-up effects on scanning behavior, a careful manipulation of saliency and task demand would be required. Alternatively, one may differentiate between predictability due to regularity in the input and due to the capacity of the system to store information, by calculating AIS while conditioning on the system’s input, as proposed in [71]. However, such an investigation is beyond the scope of this work and is subject to future investigation.

In summary, in line with previous work, our results suggest that a measure of scan path regularity, like the AIS, is an interesting candidate feature to detect changes of user states. Extending previous studies, we show that AIS is able to detect such changes also in dynamic tasks like driving. To interpret *how* changes in predictability relate to viewer behavior and states in a real-world application, additional work is necessary, which goes beyond the present experimental setup. Here, future work should aim at a more fine-grained correlation of driving behavior or other variables related to the viewer’s state to scan pattern predictability as measured by AIS.

### Inter-individual differences in scan path predictability

Applying a novel algorithm for the estimation of AIS [29, 30], we individually optimized the past time horizon considered for the estimation of scan path predictability. We demonstrated that this time horizon, that represents past fixations being predictive for the next fixation, varied between participants. Moreover, we showed that accounting for these differences had a significant impact on the interpretation of the observed scanning behavior. These results are in line with previous work showing that inter-individual differences in scan paths were better explained by a model incorporating a longer temporal horizon in the past, than only the previous last fixation ([12, 13]). Here, a clear advantage of our proposed approach to estimate AIS using state-of-the-art estimation techniques, is that the optimized past states allow for a direct interpretation of the length of the observed temporal dependencies in units of the observed time series. This was not possible in modelling approaches commonly used in previous work, i.e., the successor representation model [12], which uses a hyperparameter to represent temporal dependencies that is not directly interpretable.

Future work may further investigate whether inter-individual history lengths are task-dependent or whether inter-individual differences remain constant over tasks. Furthermore the length of the time horizon itself may be a valuable feature in predicting task success because it may reflect the ability to maintain and exploit visual information over longer time spans. Again, future work should investigate whether increases in predictability reflect increases in top-down modulation and planned behavior.

### Potential of local information-theoretic measures for scan path analysis in real-time applications

In the present work, we used local variants of both AIS and GTE that were estimated from the whole available sequence of fixations and were evaluated for periods of interest (task). We chose this approach to utilize a maximum amount of data for the estimation of scan path predictability while still being able to analyze scan path predictability locally in time. Furthermore, we believe local variants of information-theoretic measures to be a promising tool for the realization of real-time systems that make use of scan patterns, e.g., driver assistance systems [1]. Our findings are a promising step towards such an application as it suggests that local AIS and local GTE, despite being estimated from the full scan path, still allow for the detection of local changes in gaze behavior.

Using local information-theoretic measures may be advantageous in real-world scenarios, where a clear distinction into a baseline versus a task of interest is not available. Here, localized measures allow to evaluate predictability for a few fixations and even on the single-fixation level. On the other hand, the underlying probability distributions may be estimated from all available data and may even be estimated using online-learning [72]. Moreover, probability distributions may even be estimated offline to allow for an immediate use in an online system. A similar approach has been previously proposed for measuring AIS in neural systems [73]. Using all available data may allow for more robust estimates as for example estimates of averaged measures from binned data (e.g., [5]).

In sum, our results suggest that it is possible to estimate probability distributions from all available data and use them to detect local changes in gaze behavior. Future work may further explore the suitability of localized measures for online-assistance and prediction. We here present a promising step towards using entropy measures in real-time application, such as driving assistance, that typically require the detection or prediction of events on a finer temporal scale.

## Conclusion

In the present work, we evaluated a novel approach to measuring predictability of scanning behavior as a marker of changes in observer states during a dynamic driving task. We compared our proposed approach to existing measures of scan path predictability and showed that accounting for long-range temporal dependencies in scan patterns was beneficial in describing observer states. Future studies may extend the present study by validating the proposed measure in further dynamic tasks. One limitation of the current study is the small sample size. In particular, to further assess the relation between the proposed method and interindividual differences in gaze behavior, an application to larger and diverse groups of participants would be desirable. Furthermore, additional work may aim at a detailed description of how changes in scan path predictability relate to manipulations of task demand and user state, as well as interactions of both. We believe that this study is an important step towards gaining novel insights into the dynamics of viewing behavior, which may be used in gaze-based applications such as driver assistance.
